# Effect of Doll Therapy in Behavioral and Psychological Symptoms of Dementia: A Systematic Review

**DOI:** 10.3390/healthcare10030421

**Published:** 2022-02-23

**Authors:** Angela Martín-García, Ana-Isabel Corregidor-Sánchez, Virginia Fernández-Moreno, Vanesa Alcántara-Porcuna, Juan-José Criado-Álvarez

**Affiliations:** 1 Occupational Therapy Department, Altagracia Nursing Home, 28660 Madrid, Spain; angela71196@hotmail.com; 2 Faculty of Health Sciences, University of Castilla la Mancha, 45600 Talavera de la Reina, Spain; vanesa.alcantara@uclm.es; 3 HNSP–SESCAM, Health Service of Castilla la Mancha, 45600 Talavera de la Reina, Spain; vfernandezmoreno@sescam.jccm.es; 4 Institute of Sciences Health, 45006 Talavera de la Reina, Castilla la Mancha, Spain; jjcriado@jccm.es

**Keywords:** doll-therapy, dementia, behavioral and psychological symptoms of dementia

## Abstract

(1) Background: Behavioral and psychological symptoms of dementia (BPSD) are a threat for people with dementia and their caregivers. Doll therapy is a non-pharmacological person-centered therapy to promote attachment, company, and usefulness with the aim of minimizing challenging behaviors. However, the results are not clear. (2) Objective: To know the effectiveness of doll therapy in reducing behavioral and psychological symptoms of people with dementia at a moderate-severe phase. (3) Methodology: The systematic review was informed according to the criteria established by the Preferred Reporting Items for Systematic Reviews and Meta-Analyses (PRISMA) statement. Searches were conducted in eight databases: Cochrane, PubMed, Web of Science, Cinahl, Embase, Lilacs, PeDro, and Scopus before October 2021. Studies were selected when they accomplished the simple majority of Consolidated Standards of Reporting Trials (CONSORT). The risk of bias was appraised with the Cochrane Collaboration Risk of Bias Tool. The review protocol was recorded in Inplasy:1539. (4) Results: The initial search strategy showed 226 relevant studies, 7 of which met the eligibility criteria. In the included studies, a total number of 295 participants (79% female) with a mean age of 85 years were enrolled. There was found to be a reduction in challenging and aggressive behaviors, the participants were less rough and irritable, and their communication skills and emotional state were also improved. (5) Conclusion: Our findings suggest that doll therapy improves the emotional state of people with dementia, diminishes disruptive behaviors, and promotes communication. However, randomized studies with a larger sample size and higher methodological rigor are needed, as well as follow-up protocols in order to reaffirm these results.

## 1. Introduction

Dementia is one of the most common syndromes in old age with an evolution that follows an exponential pattern; it is estimated that by 2030 there will be 82 million people in the world diagnosed with dementia [1].

Alzheimer’s disease (AD) is a neurodegenerative disease of unknown etiology characterized by a progressive deterioration of memory and cognitive function and represents between 60 and 80% of dementia cases [2]. In the initial phase, it appears as temporospatial disorientation and a tendency for frequent forgetfulness, in the intermediate phase, the disorientation and memory alterations intensify and provoke difficulties in communication and the need for help to carry out daily life activities. The third phase is characterized by obstacles in orientation, walking, communicating, or recognizing close family members.

The course of AD may be affected by the appearance of psychological and cognitive symptoms of dementia (SPCD) as well. In 90% of AD cases, symptoms such as agitation, psychosis, apathy, sleep disorders, appetite changes, euphoria, irritability, aberrant motor behavior, depression, and anxiety usually appear [3]. Aggression, agitation, delirium, and erratic wandering have been identified as one of the main reasons for the overload of informal caregivers [4,5]. The impact of BPSD is so intense and overwhelming that it provokes high exhaustion, stress, anxiety, and depression in the patient, as well as in the family and caregivers, triggering institutionalization in most cases. Among professional caretakers, several studies have found that SCPD, such as agitation, erratic wandering, and aggressive episodes, may cause negative feelings and discomfort [6], causing a painful experience [7,8] and reducing their work motivation [9].

The development of programs of non-pharmacological interventions such as reminiscence therapy, music therapy, therapy with animals, or sensory stimulation therapy seems to improve the emotional wellness of people with advanced dementia. The common denominator of these techniques is based on achieving positive emotions through pleasant memories, music, or contact with pets that minimize states of anxiety or anguish, diminishing the risk of BPSD.

Doll therapy (DT) is a non-pharmacological technique with the aim to promote attachment, company, and usefulness in people with dementia to increase their wellness and minimize the appearance of challenging behaviors [10,11]. It is based on the combination of three theories: the Attachment Theory, the Transitional Object Theory, and the Person-centered Theory. The attachment theory [12] postulates the need for a human being to establish affective bonds when facing unknown situations, fear, or danger. In this way, people with dementia usually have behaviors related to attachment and fixing phenomena with their parents, looking constantly for them. DT offers the possibility to establish the affective bond needed in stress situations, thus lowering agitation.

The Transitional Object Theory [13] is based on the calming properties that certain objects may have to alleviate and diminish the anguish. Two kinds of objects have been defined: transition objects (known by the subject) [13] and precursor objects (unknown by the subject) [14]. In the case of people with dementia, the doll might be a precursor object introduced in their environment by the caregiver to give comfort and alleviate and diminish the anguish generated by the SCPD [15,16].

The Person-centered Theory was developed by Carl Rogers in 1961 [17] and places the individual at the center of care, being supported and trained to be able to collaborate with the decision-making process. Uniting this approach to positive personal workouts developed by Kitwood [18], DT can offer the possibility of developing game interactions, facilitation, and validation, converting the interactions with the doll into a positive activity and a way to connect with others.

The dolls are designed to recreate the feeling of touching, staring, dressing up, and holding a baby in their arms and can bring to the present-day older roles related to maternity and generate feelings of utility and meaning which may substitute challenging behaviors with care behaviors towards the doll. In this way, the use of dolls with a baby-like appearance (newborn dolls, reborn, or empathy dolls) generated a higher commitment from the patients in comparison with the use of stuffed and other kinds of dolls [19]. Several authors have found benefits in the use of DT, observing a decrease of negative behaviors such as agitation, aggressiveness, or erratic wandering as well as an increase in communication with the environment and independence in daily life [10,11,20]. Systematic revisions in this regard conclude that DT has positive effects on the person with dementia as long as it improves communication with the environment, alleviates the SCPD, and improves quality of life [21,22,23]. Mitchell [24,25] discovered an increase in commitment levels, communication, and reduction of anguish episodes in addition to the potential of DT to improve independence in daily life. Ng [22] concluded that people with dementia could interact in a better way with their environment after obtaining benefits from the DT. Despite these positive findings on the effect of DT, the authors warn about the scarcity of empirical studies and the need for a future investigation that includes methodologically correct clinical trials. The objective of this systematic review is double. First, the best evidence available about DT will be examined, including only clinical trials that meet most of the CONSORT (Consolidated Standards of Reporting Trials) criteria. Secondly, the relevant information for the design of treatment protocols and investigation will be extracted to allow for the establishment of clear parameters and facilitating the design of future studies of DT.

## 2. Materials and Methods

The PRISMA (Preferred Reporting Items for Systematic Reviews and Meta-Analyses) statement was employed to report this review. The protocol was registered in INPLASY:1539.

### 2.1. Bibliographic Search and Inclusion Criteria

Searches were conducted in eight databases: Cochrane, PubMed, Web of Science, Cinahl, Embase, Lilacs, PeDro, and Scopus before October 2021. No limits of date, language, or study design were established in order to increase the number of registers obtained. The search strategy was made according to the PICO (Patient, Intervention, Comparation, Outcome) methodology with the help of an expert on bibliographical resources. The search strategy used was: TITLE-ABS-KEY ((“lifelike doll” OR “baby doll” OR “doll therapy” OR “baby doll therapy” OR “doll therapy intervention” OR “doll” OR “empathy doll”) AND (“Alzheimer Disease” OR “Dementia” OR “Alzheimer” OR “Alzheimer’s” OR “Alzheimer dementia” OR “dementia sufferers” OR “nursing home resident” OR “long term care” OR “cognitive decline” OR “cognitive impairment”)). Authors were contacted to retrieve non-reported data.

The inclusion criteria were: (1) dementia diagnosis according to DSM-V; (2) people over 65 years; (3) intervention with DT; (4) clinical trials; and (5) simple majority of the CONSORT (Consolidated Standards of Reporting Trials) checklist (Appendix A). The use of several types of dolls such as empathy dolls, newborn, or reborn was accepted. The exclusion criteria were: (1) participants with severe sensory disorders that may not count due to a minimum ability to communicate or those who used dolls before the beginning of the study; (2) studies that used dolls that did not have a realistic appearance or were stuffed dolls (most of the previous studies emphasize the importance that the appearance of the doll truly resembles a real baby).

### 2.2. Data Review, Selection, and Extraction

Two independent reviewers (AMG and AICS) reviewed the titles, abstracts, and full texts. Duplicates were identified and excluded. A third reviewer (VAP) handled the disagreements. The software Covidence was used for the management and selection of the records [26]. The data extraction form was based on the Cochrane Library recommendations [27] and included information about the study (type of study, objectives, design, measures of result, and results), the participants (age, sex, kind of housing, and inclusion and exclusion criteria), the different kinds of intervention and comparisons (number of sessions, duration of each session, type of doll, and personnel involved).

### 2.3. Assessment of Risk of Bias in Individual Studies

Two independent reviewers (JJCA and VFM) were in charge of the assessment of the risk of bias of each article using the items of the Review Manager (RevMan) tool (Review Manager (RevMan) [Computer program]. Version 5.4, The Cochrane Collaboration, 2020): randomization sequence, allocation concealment, blinding of participants, blinding of assessment, attrition bias, and information bias. The risk of bias of each one of these items was determined by the following premises:

- Low risk of bias: articles in which every item obtained a low risk of bias.
- Unclear risk of bias: those studies in which one or more items had an unclear risk of bias.
- High risk of bias: studies in which one or more items had a high risk of bias.

## 3. Results

**Figure 1:**
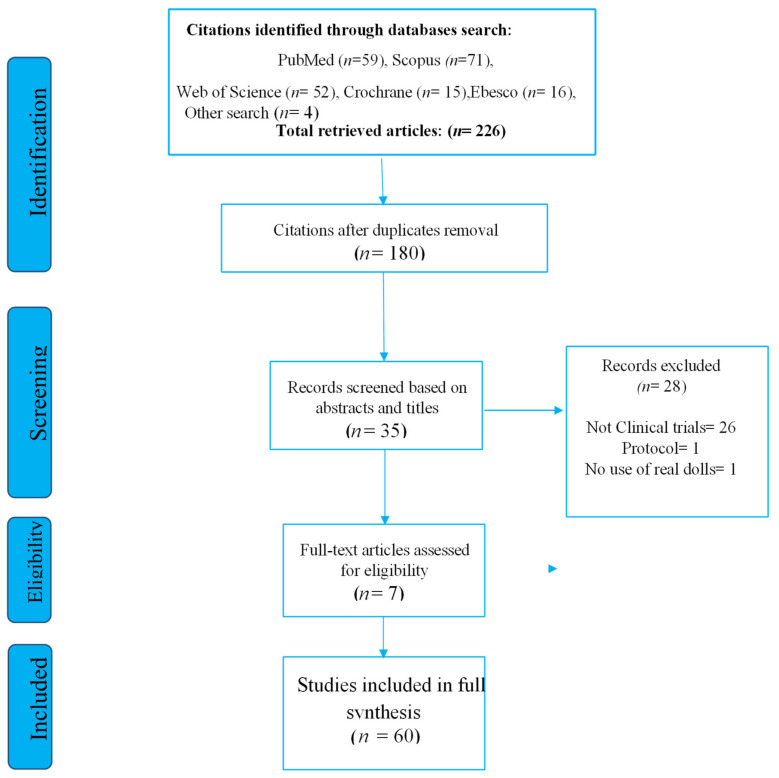
The PRISMA diagram for the records search and study selection.

The search strategy reported 226 records. Once duplicates were removed, 180 studies were screened by title and abstract according to eligibility criteria. A total of 35 articles were identified for full reading, of which 28 were excluded. Finally, seven articles were obtained for the present systematic review. The PRISMA flowchart synthesizing the study selection processes and the deletion reasons is shown in Figure 1.

### 3.1. Characteristics of the Studies and Participants Included

The articles were published between 2006 and 2020. The main objective of most of the articles was to know the efficacy and benefits of doll therapy in the neuropsychiatric symptomatology of elders with severe dementia. Three studies out of the seven were randomized clinical trials [28,29,30] one a non-randomized clinical trial [31], another an exploratory study [32] one a pilot study [33] and the last, a before-and-after study [20]. The characteristics of each study are described in Table 1.

The studies included made the assessment of the appearance of SPCD through several tests, mainly the Neuropsychiatric Inventory Questionnaire (NPI-Q), Observed Emotions Rating Scale (OERS), and Eating Behavior Scale (EBS). The quality of life in late-stage dementia (QUALID) was used as well to assess life quality.

### 3.2. Risk of Bias of Individual Studies

**Figure 2:**
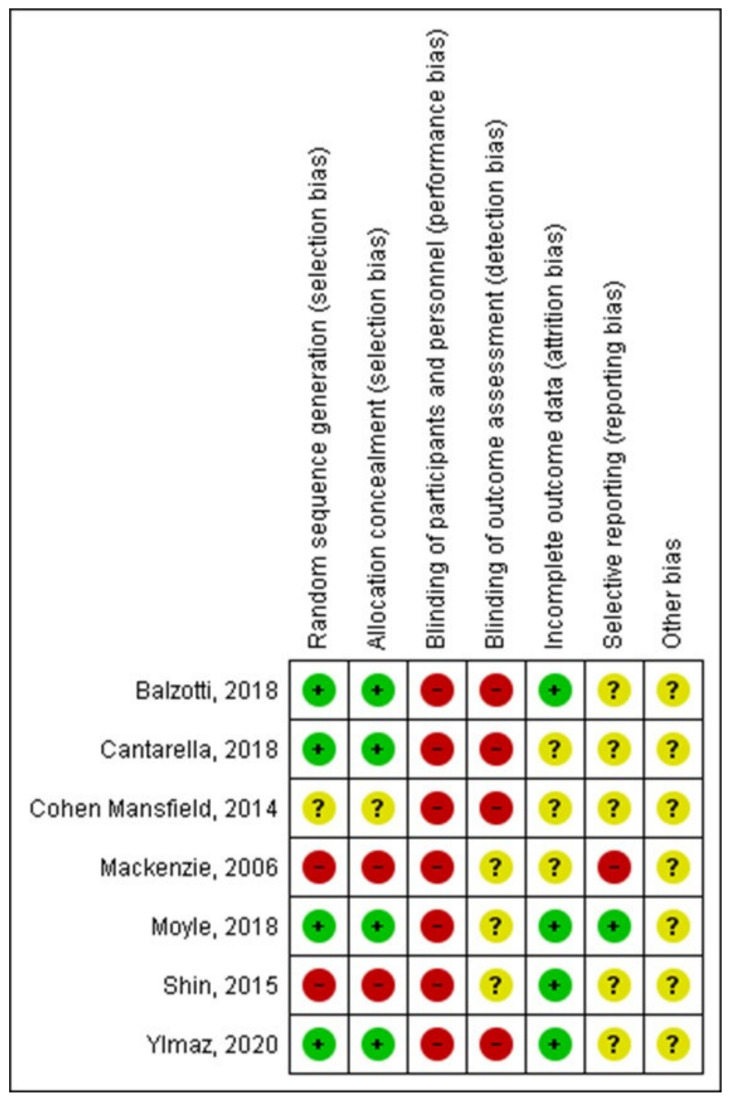
Risk of bias of studies included in the systematic review (Balzotti, 2018 [31], Cantarella, 2018 [28], Cohen Mansfield, 2014 [32], Mackenzie, 2006 [33], Moyle, 2018 [29], Shin, 2015 [20], Ylmaz, 2020 [30]).

**Figure 3:**
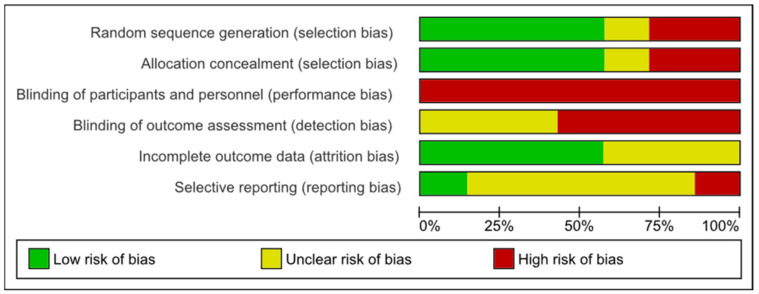
Risk of bias: systematic review authors’ judgments about each risk of bias item presented as percentages across all included trials using the Cochrane risk of bias tool (*n* = 7).

The risk of bias for each trial is summarized in Figure 2 and Figure 3. Most of the included studies completed the results with similar groups at the beginning and the end of the treatment, although this information was not clear in three of the studies [28,32,33].

Four studies had a low risk of bias in the randomization sequence and the allocation concealment. No study could blind the participants due to the characteristics of the intervention, and in four of the studies the assessment of the results was not blinded either. The report of data was only clear in one study [29].

### 3.3. Intervention with Doll Therapy

**Figure 4:**
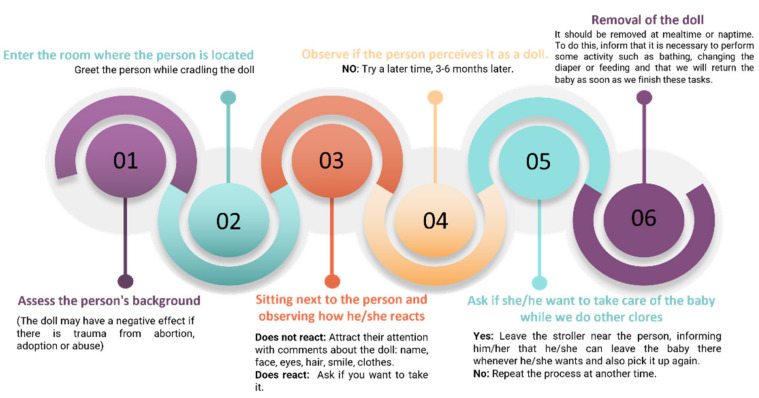
Intervention protocol with Doll Therapy.

Every study used a doll with a realistic appearance with which the person with dementia could freely interact. The duration of the sessions mainly depended on two factors: how long the participant committed to the intervention and how long were they awake. Figure 4 shows the development of an intervention protocol based on the information extracted from the included studies. This protocol is structured in six phases: starting with an evaluation of the background of the individual (phase 1), establish a way of introducing the doll and assess of the reaction of the individual in order to continue with the process (phases 2, 3, and 4); encourage the care of the doll (phase 5), and finally the removal of the doll (phase 6). The full duration of the intervention was heterogeneous, from 1 to 24 weeks.

### 3.4. Effectiveness of Doll Therapy on Behaviour

Four out of seven studies [20,29,30,32] reported a reduction in SCPD, observing the decrease of disruptive and aggressive behaviors. The participants were less agitated and irritable while holding the doll (Cantarella Mdiff: −0.025, *p* < 0.001; Shin: *t* = 16.31, *p* < 0.01; Balzotti *z* = 2.66, *p* < 0.007). They verbalized fewer swear words, fewer shouts, and fewer obsessive behaviors. Three studies [20,30,32] found a higher number of interactions with the environment, increasing social contact and verbalization (*t* = −8.41, *p* < 0.01). The episodes of erratic wandering decreased in two of the studies (Shin, *t*: −17.46, *p* < 0.001) [20,33]. Only one study [29] did not report significant evidence in reducing anxiety, agitation, and aggressiveness (Moyle, *p* < 0.88)

### 3.5. Effectiveness of Doll Therapy on Emotions

Four studies [20,29,31,32] reported benefits in the use of dolls to provide emotional support. Three of those studies found statistically significant improvements regarding the emotional component of people with dementia. Shin [20] reported statistically significant differences in positive mood and found a significant diminishment in depression (*p* < 0.01). In this way, Moyle’s work [29] had a positive effect on well-being (*p* < 0.05), and Balzotti’s study [31] reported mood changes at the third week of treatment: IC del 95% (−1.09 a 0.20) and the presence of anger (CI: −0.51 a 0.51). It also found improvements in depression (Z = 2.02, *p* = 0.04) and apathy (Z = 2.01, *p* = 0.04).

### 3.6. Effectiveness of Doll Therapy on the Basic Activities of Daily Living

Only one study evaluated the impact of DT on daily life activities. Cantarella et al. [28] studied the effect of DT on diet, one of the most problematic basic activities in people with dementia. Despite finding signs of improvement in this activity, the authors concluded that was necessary to increase the intervention time to obtain perceptible changes in the development of this activity.

## 4. Discussion

This systematic review analyzed the effectiveness of Doll Therapy to diminish the appearance of psychological and cognitive symptoms in people with dementia. This is the first updated systematic review that has selected clinical trials which met most of the CONSORT criteria and have been reported according to the PRISMA statement.

Previous systematic reviews [22,24] have included qualitative studies that were mainly narratives of professionals about their impressions about the effect of DT, not the group randomization measurement of the effect with valid evaluation tools. This led to reporting conclusions that could move away from real effectiveness due to methodological bias. To avoid this, our systematic review collected information from studies that methodologically met the randomization and objective evaluation of results criteria.

DT is a technique that started to be used in the 1980s. From its beginnings, it has provoked contrary opinions and an ethical dilemma in the professionals working with people with dementia. Several authors [11,34,35] express their concern about the ethical conflicts that may derive from this technique, considering it a practice that infantilizes and could potentially undermine the dignity of the person. On the other hand, there are other authors that defend the use of this technique, claiming the benefits of its applications [10,25,35]. For our part, the results obtained in this systematic review report that DT produces positive changes and statistically significant results in the diminishing of disruptive behaviors such as erratic wandering, aggressiveness, agitation, and negative verbalization. We have also found that most of the included studies report improvements in the emotional component of people with dementia, resulting in fewer episodes of suffering, and witnessing more positive moods. These changes may be due to the interaction and meaning that the person with dementia has with the doll, corroborating the emotional benefits generated by attachment and person-centered attention found in previous studies [25,36,37].

Related to the time of intervention, it was found that a prolonged duration contributes to the obtention of positive results, even producing changes in food intake. The study developed by Cohen [32] found that a 6-month intervention allows for the development of an initial test phase and familiarization with the doll in people with dementia, as well as their families; and a later phase in which the treatment was implemented to obtain more effective results on the behaviors of rejection towards the intervention and overall behavioral symptoms. Moreover, a prolonged intervention allows for a higher acceptance of DT, since caregivers and families can observe the benefits in a more complete way. On the other hand, it is also important to plan post-intervention follow-up in order to observe if the participants maintain the changes in behavior after applying the therapy. Most of the studies of this review do not include any follow-up after the end of the intervention with dolls.

Nevertheless, the interpretation of this data should be taken with caution and be considered in the context of several methodological problems. The randomization sequence and the concealment were only clear in half of the studies, and the blinding of the evaluation was not clear in any study, so the obtained results can lead to higher estimations than the real effect of DT over psychological and behavioral symptoms of dementia. Previous reviews [21,22] found similar methodological limitations to DT and that is why we suggest further studies that might design protocols that control possible confusion factors, as well as the planification during and after the intervention.

In relation to daily life activities, only one article [29] studied the impact of DT on the performance of daily life activities, finding benefits at the time of feeding.

Related to the limitations of this review, it is probable that not all the studies could have been identified, despite using exhaustive search strategies. The methodological demand of inclusion criteria is the reason for the small number of studies included in the review; this might be a limitation, but ensures the reliability of the obtained evidence. Additionally, the included studies had a small sample size, which could have conditioned the effect of the intervention. Furthermore, it has been not possible to know the lasting effect of DT on the psychological and behavioral symptoms of dementia, given the absence of subsequent follow-up in most of the studies.

The results obtained in this systematic review have important implications for socio-sanitary professionals that provide care to people with dementia, as it reports the benefits that DT entails in the improvement of behavioral symptoms and mood. At the same time, guidelines are provided for the implementation of this type of non-pharmacological therapy which can be summarized into four items:

- Doll therapy reduces psychological and behavioral symptoms of dementia.
- It is beneficial to follow a six phase protocol for treatment (evaluation, introduction of the doll, assessment of the reaction, presentation of the doll, care of the doll, and the removal of the doll).
- The prolonged duration of doll therapy allows for achieving more benefits.
- Future studies must include the randomization and the blinding of the assessment to increase the methodological quality.

## 5. Conclusions

Our findings suggest that doll therapy improved the emotional state, diminished disturbing behaviors, and enhanced communication with the environment in dementia patients. However, randomized studies with a greater sample size and methodological rigor are needed, as well as follow-up protocols to reaffirm these results.

## Figures and Tables

**Table 1:** Characteristics of the studies included in the systematic review.

Author	Type of Study and Participants				Characteristics of the Intervention of Doll Therapy					Results
	*n*	Study	Age (Yrs)	Sex	Inclusion Criteria	Emplacement	Experimental Group	Control Group	Outcome Measure	
**Moyle**, **2018** [29]	35	RCT	<65 years	Female	<65 years, dementia diagnosis; documented history (in last four weeks) of anxiety, agitation, or aggressiveness.	Residents were recruited from five LTC facilities located within a 60 km radius of the Brisbane Central Business District (Queensland, Australia)	Doll Therapy	Usual treatment	Mini Mental CMAI-SF; OERS	Clinically significant improvements in the well-being of residents in comparison with the usual care, but there were no improvements in anxiety, agitation, and aggressiveness.
**Balzoti** **2018** [31]	30	Non-randomized clinical trial	<65 years	25 Females 10 Males	Severe to low cognitive impairment, behavioral disorders, <65 years, dementia.	Residenza Sociosanitaria Assistenziale per Anziani “Storelli” in Bisceglie (Italia)	1. Doll therapy 2. Gestural-verbal treatment	No intervention	NPI-Q	Doll therapy was effective for the reduction of agitated and irritable behaviors. No changes in apathy were found.
**Cantarella**, **2018** [28]	32	RCT	<70 years	26 Females 6 Males	Punctuation of ≥5 in the Short Portable Mental Status Questionnaire; <70 years; dementia diagnosis, post-traumatic stress disorder according to doctors; no participation in other non-pharmacological interventions before or during the study; without severe sensorial or perceptive deficiencies or ongoing mourning; and the capacity of understanding easy messages and producing sentences.	Residential facilities	Doll Therapy	Hand-warmer	SPMSQ EBS	Significative reduction in post-traumatic stress disorder, relief of negative feelings, fulfilling of attachment needs, and the reduction of the feeling of loneliness. Several aspects that influence food intake, such as anguish, improved but not enough to improve the eating behavior.
**Yilmaz**, **2020** [30]	29	RCT	82–89 years	15 Females 14 Males	Moderate-severe dementia, motor abilities needed to hold and caress a doll, adequate visual and auditive functions, and ability to communicate in Turkish.	A. KadirU¨ c¸yıldız, elder facility	Doll Therapy	No intervention	SMMSE CMAI NPI-Q	Statistically significant improvements in agitation and behavior problems. Cognition did not improve.
**Shin**, **2015** [20]	62	pre-post	82.4 years	86.3% Females 74.5% Males	Slight-severe cognitive impairment, three months residing in the nursing home.	Korea nursing home	Doll Therapy	-	SMMSE QUALID	Statistically significant decrease in the use of swear words, shouts, aggressive episodes, and less obsessive behaviors. Erratic wandering episodes were reduced as well. There were found positive changes in moods and physical appearance, a decrease of depression, and an increment of the interactions with other individuals, but without significant differences.
**Mackenzie**, **2006** [33]	14	Pilot study	75–94 years	12 Females 2 Males	-	Nursing home	Doll Therapy	-	Ad hoc questionnaire	Increase in social interaction. The participants seemed to be happier and less agitated. They were also more receptive towards personal care activities; erratic wandering episodes were reduced.
**Cohen-Mansfield**, **2014** [32]	93	Exploratory study	85.9 years	73% Females	Three months residing at a nursing home, behavior disorders, <60, dementia diagnosis.	Maryland nursing home	Doll Therapy	-	MMSE CAR LMBS	There was a rejection of Doll Therapy; it is associated with a low social level. In spite of this, it was one of the most used therapies and obtained a relatively high rate for the impact on the behavioral symptoms.

CAR: Change Assessment Rating; CMAI-SF: Cohen-Mansfield Agitation Inventory-Short Form; EBS: Eating Behavior Scale; LMBS: Lawton’s Modified Behavior Stream; LTC: Long term care; MMSE: Mini-Mental Status Examination; NPI-Q: Neuropsychiatric Inventory Questionnaire; OERS: Observed Emotion Rating Scale; QUALID: quality of life in late-stage dementia; SMMSE: Standardized Mini-Mental State Examination; SPMSQ: short portable mental status questionnaire; RCT: randomized controlled trial.

## Appendix A

The appendix is an optional section that can contain the methodological analysis of the studies according to the checklist Consolidated Standards of Reporting Trials (CONSORT).

	**Balzotti** [32]	**Cantarella** [29]	**Cohen-Mansfield, ** [28]	**Mackenzie, ** [30]	**Moyle,** [31]	**Shin, ** [20]	**Yilmaz, ** [36]
**1A**	NO	NO	NO	NO	X	NO	X
**1B**	X	X	X	NO	X	NO	X
**2A**	X	X	X	X	X	X	X
**2B**	X	X	X	X	X	NO	X
**3A**	NO	NO	NO	X	X	X	X
**3B**	NO	NO	NO	NO	NO	NO	NO
**4A**	X	X	X	NO	X	X	X
**4B**	X	X	X	X	X	X	X
**5**	X	X	X	X	X	NO	X
**6A**	X	X	X	NO	X	X	X
**6B**	X	X	X	NO	NO	NO	NO
**7A**	NO	NO	NO	NO	X	X	X
**7B**	NO	X	NO	NO	X	NO	NO
**8A**	NO	NO	X	NO	X	NO	X
**8B**	NO	NO	NO	NO	NO	NO	NO
**9**	NO	NO	NO	NO	X	NO	X
**10**	NO	NO	NO	NO	X	NO	NO
**11A**	X	NO	NO	NO	X	N	X
**11B**	X	X	NO	NO	NO	NO	NO
**12A**	X	X	X	NO	X	NO	X
**12B**	X	NO	NO	NO	X	NO	NO
**13A**	NO	NO	X	NO	X	NO	X
**13B**	X	X	NO	NO	X	NO	X
**14A**	NO	NO	NO	NO	NO	X	NO
**14B**	X	X	X	X	X	X	X
**15**	X	X	NO	X	X	NO	X
**16**	X	X	NO	X	X	NO	X
**17A**	NO	X	X	NO	X	NO	X
**17B**	NO	X	NO	NO	X	NO	X
**18**	X	NO	X	NO	NO	NO	NO
**19**	NO	NO	X	X	X	X	X
**20**	X	X	X	X	X	X	X
**21**	X	NO	NO	NO	X	X	NO
**22**	X	X	X	X	X	X	X
**23**	X	X	X	X	X	X	X
**24**	NO	NO	NO	NO	NO	NO	NO
**25**	NO	NO	X	NO	X	NO	X

## References

[1] OMS|La Demencia: Una Prioridad Para La Salud Pública. http://www.who.int/mental_health/neurology/dementia/es/.

[2] Garre-Olmo J. (2018). Epidemiology of Alzheimer’s disease and other dementias. Rev. Neurol..

[3] Radue R., Walaszek A., Asthana S. (2019). Neuropsychiatric Symptoms in Dementia. Handb. Clin. Neurol..

[4] Etters L., Goodall D., Harrison B.E. (2008). Caregiver Burden among Dementia Patient Caregivers: A Review of the Literature. J. Am. Acad. Nurse Pract..

[5] Chiao C.-Y., Wu H.-S., Hsiao C.-Y. (2015). Caregiver Burden for Informal Caregivers of Patients with Dementia: A Systematic Review. Int. Nurs. Rev..

[6] Holst A., Skär L. (2017). Formal Caregivers’ Experiences of Aggressive Behaviour in Older People Living with Dementia in Nursing Homes: A Systematic Review. Int. J. Older People Nurs..

[7] Miyamoto Y., Tachimori H., Ito H. (2010). Formal Caregiver Burden in Dementia: Impact of Behavioral and Psychological Symptoms of Dementia and Activities of Daily Living. Geriatr. Nur..

[8] Song J.-J. (2019). Virtual Reality for Vestibular Rehabilitation. Clin. Exp. Otorhinolaryngol..

[9] Lim D.Y. (2015). Coping with dementia related behavior problems of the elderly and care providers. J. Korea Acad.-Ind. Coop. Soc..

[10] Bisiani L., Angus J. (2013). Doll Therapy: A Therapeutic Means to Meet Past Attachment Needs and Diminish Behaviours of Concern in a Person Living with Dementia—A Case Study Approach. Dement. Lond. Engl..

[11] Mitchell G., O’Donnell H. (2013). The Therapeutic Use of Doll Therapy in Dementia. Br. J. Nurs..

[12] Pezzati R., Molteni V., Bani M., Settanta C., Di Maggio M.G., Villa I., Poletti B., Ardito R.B. (2014). Can Doll Therapy Preserve or Promote Attachment in People with Cognitive, Behavioral, and Emotional Problems? A Pilot Study in Institutionalized Patients with Dementia. Front. Psychol..

[13] Winnicott D.W. (1974). Objetos transicionales. Realidad Y Juego.

[14] Gaddini R. (1979). The Relationship of Reactions to Illness to Developmental Stages. Bibl. Psychiatr..

[15] Stephens A., Cheston R., Gleeson K. (2013). An Exploration into the Relationships People with Dementia Have with Physical Objects: An Ethnographic Study. Dementia.

[16] LoboPrabhu S., Molinari V., Lomax J. (2007). The Transitional Object in Dementia: Clinical Implications. Int. J. Appl. Psychoanal. Stud..

[17] Rogers C.R. (1961). The Place of the Person in the New World of the Behavioral Sciences. Pers. Guid. J..

[18] Kitwood T. (1998). Professional and Moral Development for Care Work: Some Observations on the Process. J. Moral Educ..

[19] Tamura T., Nakajima K., Nambu M., Nakamura K., Yonemitsu S., Itoh A., Higashi Y., Fujimoto T., Uno H. (2001). Baby Dolls as Therapeutic Tools for Severe Dementia Patients. Gerontechnology.

[20] Shin J.H. (2015). Doll Therapy: An Intervention for Nursing Home Residents with Dementia. J. Psychosoc. Nurs. Ment. Health Serv..

[21] Chinnaswamy K., DeMarco D.M., Grossberg G.T. (2021). Doll Therapy in Dementia: Facts and Controversies. Ann. Clin. Psychiatry.

[22] Ng Q.X., Ho C.Y.X., Koh S.S.H., Tan W.C., Chan H.W. (2017). Doll Therapy for Dementia Sufferers: A Systematic Review. Complement. Ther. Clin. Pract..

[23] Cai X., Zhou L., Han P., Deng X., Zhu H., Fang F., Zhang Z. (2021). Narrative Review: Recent Advances in Doll Therapy for Alzheimer’s Disease. Ann. Palliat. Med..

[24] Mitchell G., McCormack B., McCance T. (2016). Therapeutic Use of Dolls for People Living with Dementia: A Critical Review of the Literature. Dementia.

[25] Mitchell G. (2014). Use of Doll Therapy for People with Dementia: An Overview. Nurs. Older People.

[26] Covidence—Better Systematic Review Management. https://www.covidence.org/.

[27] Chandler J., Higgins J.P., Deeks J.J., Davenport C., Clarke M.J. (2019). Cochrane Handbook for Systematic Reviews of Interventions.

[28] Cantarella A., Borella E., Faggian S., Navuzzi A., De Beni R. (2018). Using Dolls for Therapeutic Purposes: A Study on Nursing Home Residents with Severe Dementia. Int. J. Geriatr. Psychiatry.

[29] Moyle W., Murfield J., Jones C., Beattie E., Draper B., Ownsworth T. (2019). Can Lifelike Baby Dolls Reduce Symptoms of Anxiety, Agitation, or Aggression for People with Dementia in Long-Term Care? Findings from a Pilot Randomised Controlled Trial. Aging Ment. Health.

[30] Yilmaz C.K., Aşiret G.D. (2021). The Effect of Doll Therapy on Agitation and Cognitive State in Institutionalized Patients With Moderate-to-Severe Dementia: A Randomized Controlled Study. J. Geriatr. Psychiatry Neurol..

[31] Balzotti A., Filograsso M., Altamura C., Fairfield B., Bellomo A., Daddato F., Vacca R.A., Altamura M. (2019). Comparison of the Efficacy of Gesture-Verbal Treatment and Doll Therapy for Managing Neuropsychiatric Symptoms in Older Patients with Dementia. Int. J. Geriatr. Psychiatry.

[32] Cohen-Mansfield J., Marx M.S., Dakheel-Ali M., Thein K. (2015). The Use and Utility of Specific Nonpharmacological Interventions for Behavioral Symptoms in Dementia: An Exploratory Study. Am. J. Geriatr. Psychiatry.

[33] Mackenzie L., James I.A., Morse R., Mukaetova-Ladinska E., Reichelt F.K. (2006). A Pilot Study on the Use of Dolls for People with Dementia. Age Ageing.

[34] Higgins P. (2010). Using Dolls to Enhance the Wellbeing of People with Dementia in Residential Care. Nurs. Times.

[35] Salari S.M. (2002). Intergenerational Partnerships in Adult Day Centers: Importance of Age-Appropriate Environments and Behaviors. Gerontologist.

[36] Managing Challenging Behaviors in Patients with Dementia: The Use of Therapy Dolls|Article|NursingCenter. https://www.nursingcenter.com/journalarticle?Article_ID=5460106&Journal_ID=417221&Issue_ID=5459955.

[37] Santagata F., Massaia M., D’Amelio P. (2021). The Doll Therapy as a First Line Treatment for Behavioral and Psychologic Symptoms of Dementia in Nursing Homes Residents: A Randomized, Controlled Study. BMC Geriatr..

